# Education and clinical pharmacist-led management strategies for the risk and prophylaxis of venous thromboembolism in general surgery

**DOI:** 10.1186/s12959-023-00530-2

**Published:** 2023-08-09

**Authors:** Zeynep Karaburç Kiracı, Nadir Yalçın, Ömer Cennet, Kutay Demirkan, Kaya Yorgancı

**Affiliations:** 1 Department of Clinical Pharmacy, Faculty of Pharmacy, Hacettepe University, Ankara, 06230 Türkiye; 2 Department of General Surgery, Faculty of Medicine, Hacettepe University, Ankara, 06230 Türkiye

**Keywords:** Venous thromboembolism, Prophylaxis, Thromboprophylaxis, Clinical pharmacist, General surgery

## Abstract

**Background:**

Despite the risks of venous thromboembolism (VTE) in surgical patients are well defined, primary thromboprophylaxis (TP) can be neglected. The aim of this study was to evaluate the risk of VTE and appropriateness of TP and to assess the effects of education and clinical pharmacy (CP) services.

**Methods:**

This study was conducted in a total of 3 periods (*n* = 800): pre-education (*n* = 340), post-education (*n* = 269) and CP intervention period (*n* = 191) and the risk of VTE and the appropriateness of TP were evaluated. At the end of pre-education period, patients were re-evaluated after education was given about the guidelines on TP and an educative poster was posted in the services (post-education period). During the CP intervention period, the CP made recommendations in terms of optimal TP use to the physicians in charge.

**Results:**

While there was no significant difference in the optimal TP rate administered to the patients before and after education (138/340, 40.6% vs. 122/269, 45.4%; *p* = 0.238); this rate was increased to 113/191 (59.2%) in the CP intervention period (*p* = 0.004). High-risk patients who received one type of TP constituted the majority of patients who did not receive optimal TP. While the ratio of high-risk patients undergoing a single type of TP in the pre- and post-education periods (104/340, 30.6% vs. 83/269, 30.9%), was similar (*p* = 0.819); with the CP interventions, this rate was reduced to 35/191 (18.3%) (*p* = 0.001).

**Conclusion:**

Even though education has positive influence on surgeons, the implementation of CP practices is more effective especially in terms of maintaining optimal TP.

## Background

Thromboprophylaxis (TP) is recommended in risky hospitalized patients. In the absence of TP, the incidence of asymptomatic deep vein thrombosis (DVT) is 15–80% in surgical and trauma patients and 10–40% in medical patients. Diagnosis and treatment of both the disease itself and its complications (post-thrombotic syndrome, chronic venous insufficiency, pulmonary thromboembolism, and chronic thromboembolic pulmonary hypertension) are difficult and high-cost [1].

VTE is the most important and primary cause of mortality after surgical procedures. Despite the risks of VTE in surgical patients are well defined, primary TP may be neglected in surgical patients in the worldwide. In a multicenter international study, it was observed that appropriate TP was provided to only 58.5% of surgical patients [2].

The degree of VTE risk varies according to the type and duration of surgery, immobilization status of the patient and the presence of other VTE risks in the patient. Various models have been developed to facilitate prediction of the degree of VTE risk. The most common and widely used model is the Caprini Risk Assessment Model (RAM), which has been validated and is easy to apply in clinical practice. Caprini RAM was first developed in 1991 and it was updated in 2013 [3, 4]. In Caprini RAM, VTE risks are categorized in four groups: Very low (Caprini score: 0, early mobilization is sufficient and no additional prophylaxis is required), low (Caprini score: 1–2, mechanical prophylaxis is recommended), moderate (Caprini score: 3–4, mechanical or pharmacological prophylaxis is recommended) and high (Caprini score: ≥ 5, both mechanical and pharmacological prophylaxis are recommended) VTE risk. Patients at intermediate or high risk of VTE with a high risk of bleeding require mechanical prophylaxis until the risk of bleeding decreases. Low molecular weight heparin (LMWH) or standard heparin (SH) is generally preferred for pharmacological prophylaxis. Fondaparinux and aspirin are recommended only when heparin is contraindicated [5]. Prophylaxis is usually continued for 7–10 days until the patient is mobilized and discharged from the hospital. Prolonged prophylaxis (> 28 days) is recommended in patients undergoing abdominal-pelvic cancer surgery and at high risk of VTE [6].

The aim of this study was to evaluate the risk of VTE and appropriateness of TP use in hospitalized patients in the general surgery wards and to examine the contribution of education and clinical pharmacist for improvement.

## Methods

**Fig. 1:**
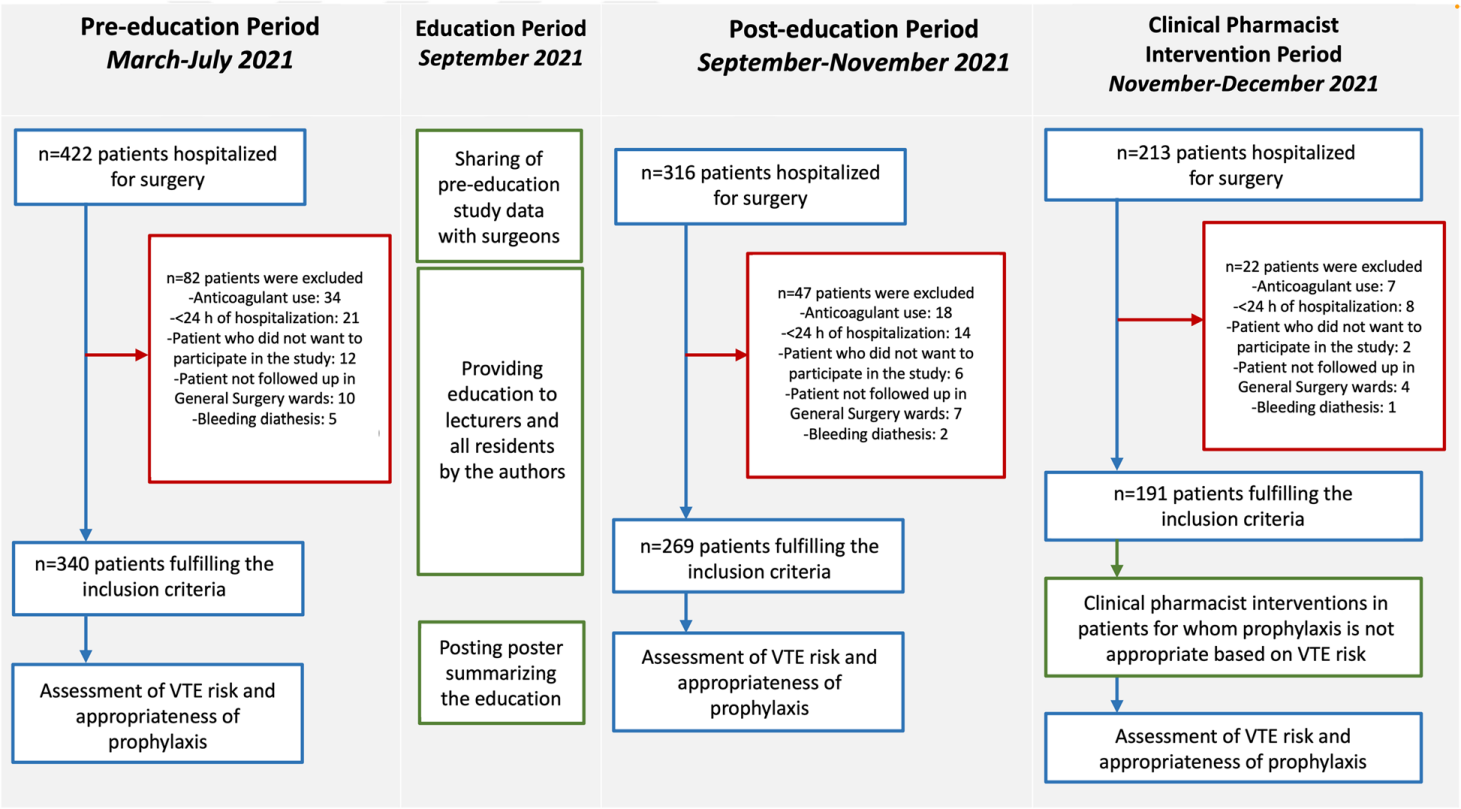
Study flow chart

This is a single-center prospective study conducted in a university hospital in 3 periods between March 2021 and December 2021 (Fig. 1). Patients aged 18 years and older who had a hospitalization plan of at least 24 h, were hospitalized with an indication for surgery, were conscious preoperatively or were accompanied by a first-degree relative were included in the study. Patients who were on any anticoagulant drug, had bleeding tendency, were not followed up in general surgery wards, had a hospitalization period of less than 24 h and did not give consent to participate in the study were excluded from the study.

In the first period of the study (pre-education period), the risk of VTE and the appropriateness of prophylaxis in patients hospitalized with an indication for surgery were assessed by clinical pharmacist according to American College of Chest Physicians (ACCP) 2012 guideline [7]. At the end of the first period, online information (on analysis of their anonymized current practice) and education (on Caprini RAM, VTE risks, indications, choice, doses, frequency and duration of TP according to ACCP 2012 guideline) were provided to all surgeons. In addition, immediately after the information and education was given, 80 × 100 cm posters containing the summary of the education were posted in visible areas in all general surgery wards, including the general surgery intensive care unit, burn unit, and in the resident rooms, and were not removed until the end of the study. In the second period of the study (post-education period), the risk of VTE and the appropriateness of prophylaxis in patients hospitalized with an indication for surgery were re-evaluated and data were compared in order to determine whether there was an improvement after providing information and education. In the last period of the study, in addition to assessment of the appropriateness of VTE risk and prophylaxis, clinical pharmacist recommendations were made in line with ACCP 2012 guideline to the physicians in charge. In our study, the administered TP method was considered appropriate in patients who required mechanical prophylaxis and who received compression stockings.

Patients who were partially appropriate for the administered TP were determined as follows: Patients at high risk of VTE without contraindications for pharmacological prophylaxis were administered a single type of prophylaxis (mechanical or pharmacological prophylaxis), and patients at moderate risk of VTE were administered to receive both pharmacological and mechanical prophylaxis. Patients for whom the select of TP method was not appropriate were defined as those at low, moderate or high risk for VTE and who do not receive any prophylaxis. In all periods, the incidence of VTE and bleeding in the post-operative 30-day follow-up were also evaluated.

In this study, Caprini RAM 2013 was used [5]. Caprini RAM 2013 has not been tested in validation studies, but it differs from Caprini RAM 2005 in that it includes additional risk factors shown to be associated with thrombosis in the literature. Body mass index (BMI) > 40 kg/m2, smoking, diabetes mellitus with insulin use, chemotherapy, blood transfusion, > 2 h of surgery time were added risk factors and each parameter is determined as 1 point in the scoring [3].

### Statistical analysis

Since there is no precedent study in the literature, the sample size could not be calculated. Instead, the power was calculated considering the data obtained in the study. With an effect size of 0.147, 95% power, 5% margin of error, the power of the 3-group study was found to be 95.06% (G*Power Version 3.0.10).

As descriptive statistics, mean and standard deviation or median and minimum–maximum values for numerical variables and number and percentage values for categorical variables were given. Normality assumption, one of the parametric test assumptions, was analyzed by Kolmogorov–Smirnov test and graphical representations. In the comparison of numerical data, Student T Test was used for normally distributed data and Mann Whitney U test was used for non-normally distributed data. Chi-Square test was used to compare the ratios. In analyzing the change over time, the significance test of the difference between two pairs or Wilcoxon test was used. The relationship between numerical variables was analyzed using the appropriate correlation test (Pearson or Spearman). Regression analysis, which is a statistical analysis used to quantify the relationship between a criterion variable and one or more predictor variables, mainly aims to determine the nature of the relationship between variables. In our study, the *p* value between the pre-training period and the post-training period was defined as p1 and the *p* value between the post-training period and the clinical pharmacist intervention period was defined as p2. For all tests, *p* < 0.05 was considered statistically significant. All analyses were carried out in the *IBM SPSS Statistics Version 23* software.

### Ethics approval

The study was approved by the Local Ethics Committee (decision no: 2021/07–52).

## Results

### Demographics

A total of 800 patients, 340 in the pre-education period, 269 in the post-education period, and 191 in the clinical pharmacist-intervention period, were included in the study. It was found that the length of hospital stay (LOS) in the pre-education period was significantly longer than the post-education period [median (range): 5 (1–275) and 4 (1–288) days; *p* = 0.023] (Table 1).

**Table 1:** Demographic characteristics

**Variables**	**Pre-education period (*n* = 340)**	**Post-education period (*n* = 269)**	**Intervention period (*n* = 191)**	**Total (*n* = 800)**	**p1**	**p2**	**p3**
Age (year), mean (SD)	51.1 (14.73)	50.81 (15.4)	54.82 (15.05)	51.9 (15.13)	0.780	**0.004**	**0.003**
Gender (female), n (%)	182 (53.5)	152 (56.5)	118 (61.8)	452 (56.5)	0.464	0.258	0.085
BMI (kg/m^2^), mean (SD)	27.37 (5.05)	27.78 (5.52)	28.13 (5.15)	27.7 (5.24)	0.609	0.372	0.195
GFR < 30 mL/min, n (%)	3 (0.9)	1 (0.4)	-	4 (0.5)	0.634	1.000	0.556
Emergency surgery, n (%)	18 (5.3)	33 (12.3)	4 (2.1)	55 (6.9)	**0.003**	** < 0.001**	0.110
Abdominal-pelvic cancer surgery, n (%)	66 (19.4)	32 (11.9)	26 (13.6)	124 (15.5)	**0.012**	0.585	0.125
LOS (day), median (min–max)	5 (1–275)	4 (1–288)	4 (2–42)	4 (1–288)	**0.023**	0.970	0.012
Caprini RAM, median (min–max)	6 (1–17)	5 (1–14)	6 (1–12)	6 (1–17)	0.548	0.154	0.301
Type of surgery, n (%)							
Colorectal surgery	72 (20.7)	58 (21.3)	30 (15.2)	160 (20.0)	0.908	0.116	0.125
*Colon*	17	15	7	39	0.894	0.344	0.622
*Rectum*	18	13	7	38	0.943	0.709	0.524
*Anus*	37	30	16	83	0.916	0.412	0.439
Hepatobiliary Surgery	69 (19.9)	44 (16.2)	44 (22.3)	158 (19.3)	0.215	0.073	0.587
Hernia Surgery	58 (16.7)	48 (17.6)	40 (20.3)	146 (17.8)	0.800	0.405	0.181
Breast Surgery	57 (16.4)	40 (14.7)	39 (19.8)	136 (16.6)	0.526	0.120	0.336
Endocrine Surgery	35 (10.0)	32 (11.4)	22 (11.2)	89 (10.9)	0.455	1.000	0.585
*Thyroid*	24	20	18	62	0.984	0.554	0.338
*Parathyroid*	9	10	4	23	0.603	0.414	0.778
*Adrenal*	2	2	0	4	1.000	0.513	0.538
Lower GI Surgery	16 (4.6)	25 (9.1)	10 (5.1)	51 (6.2)	**0.037**	0.150	0.746
Upper GI Surgery	21 (6.0)	13 (4.8)	6 (3.0)	40 (4.9)	0.590	0.509	0.186
Others	19 (5.5)	13 (4.8)	6 (3.0)	38 (4.6)	0.816	0.509	0.287
Total^a^	347 (100)	272 (100)	197 (100)	818 (100)	0.706	0.121	0.228

*BMI* Body mass index, *GFR* Glomerular filtration rate, *LOS* Length of hospital stay, *RAM* Risk assessment model, *GI* Gastrointestinal

p1: *P* value between pre-training and post-training period

p2: *p* value between post-training and the clinical pharmacist-intervention period

p3: *p* value between pre-training and the clinical pharmacist-intervention period

^a^Since there may be more than one type of surgery in a patient, all types of surgery were summed

According to the Caprini RAM, BMI > 25 kg/m^2^ (67.1%), history of major surgery (64.0%), 41–60 years of age (45.6%), history of cancer (45.4%), and length of surgery over 2 h (25.4%) were detected as most common risk factors for VTE. According to the total score, 5.3% of the patients were at low-risk, 24.5% moderate-risk, and 70.2% high-risk. Also, there was no statistically significant difference between the groups (*p* > 0.05) (Table 2).

**Table 2:** Comparison of patients’ data according to the Caprini risk assessment model

**Variables**	**Pre-education period (*n* = 340), n (%)**	**Post-education period (*n* = 269), n (%)**	**Intervention period (*n* = 191), n (%)**	**Total (*n* = 800), n (%)**	**p1**	**p2**	**p3**
Body mass index > 25 kg/m2	231 (67.9)	180 (66.9)	126 (66.0)	537 (67.1)	> 0.05	> 0.05	> 0.05
Major surgery	205 (60.3)	176 (65.4)	131 (68.6)	512 (64.0)	> 0.05	> 0.05	> 0.05
41–60 years of age	158 (46.5)	126 (46.8)	81 (42.4)	365 (45.6)	> 0.05	> 0.05	> 0.05
History of cancer	157 (46.2)	104 (38.7)	86 (45.0)	347 (43.4)	> 0.05	> 0.05	> 0.05
Length of surgery over 2 h	80 (23.4)	77 (28.6)	46 (24.1)	203 (25.4)	> 0.05	> 0.05	> 0.05
Smoking within the last month	85 (25.0)	70 (26.0)	45 (23.6)	200 (25.0)	> 0.05	> 0.05	> 0.05
Laparoscopic surgery	81 (23.8)	62 (23.0)	50 (26.2)	193 (24.1)	> 0.05	> 0.05	> 0.05
61–74 years of age	86 (25.3)	56 (20.8)	61 (31.9)	203 (25.4)	> 0.05	**0.007**	> 0.05
Minor surgery	54 (15.9)	31 (11.5)	10 (5.2)	95 (11.9)	> 0.05	**0.030**	**0.002**
Varicose veins	41 (12.1)	29 (10.8)	20 (10.5)	90 (11.2)	> 0.05	> 0.05	> 0.05
Serious infection	26 (7.6)	21 (7.8)	9 (4.7)	56 (7.0)	> 0.05	> 0.05	> 0.05
Swollen legs	20 (5.8)	24 (8.9)	10 (5.2)	54 (6.8)	> 0.05	> 0.05	> 0.05
Chemotherapy	28 (8.2)	14 (5.2)	6 (3.1)	48 (6.0)	> 0.05	> 0.05	> 0.05
Diabetes requiring insulin	22 (6.5)	10 (3.7)	10 (5.2)	42 (5.3)	> 0.05	> 0.05	> 0.05
History of unexplained stillborn	18 (5.2)	13 (4.8)	10 (5.2)	41 (5.1)	> 0.05	> 0.05	> 0.05
≥ 75 years of age	11 (3.2)	18 (6.7)	12 (6.3)	41 (5.1)	> 0.05	> 0.05	> 0.05
Family history of blood clots	13 (3.8)	13 (4.8)	7 (3.7)	33 (4.1)	> 0.05	> 0.05	> 0.05
Lung disease	10 (2.9)	14 (5.2)	7 (3.7)	31 (3.9)	> 0.05	> 0.05	> 0.05
Body mass index > 40 kg/m^2^	8 (2.4)	8 (3.0)	5 (2.6)	21 (2.6)	> 0.05	> 0.05	> 0.05
History of venous thromboembolism	6 (1.8)	6 (2.2)	4 (2.1)	16 (2.0)	> 0.05	> 0.05	> 0.05
Current use of birth control or hormone replacement therapy	8 (2.3)	4 (1.4)	7 (3.7)	19 (2.4)	> 0.05	> 0.05	> 0.05
Confined to bed for 72 h or more	6 (1.8)	1 (0.4)	-	7 (0.8)	> 0.05	-	-
Congestive heart failure	1 (0.3)	5 (1.9)	-	6 (0.7)	> 0.05	-	-
Recurrent spontaneous abortion (3 or more)	4 (1.1)	1 (0.3)	1 (0.5)	6 (0.7)	> 0.05	> 0.05	> 0.05
History of inflammatory bowel disease	1 (0.3)	2 (0.7)	-	3 (0.4)	> 0.05	-	-
Blood transfusion(s)	-	3 (1.1)	-	3 (0.4)	-	-	-
Pregnant or had a baby within the last month	1 (0.5)	1 (0.6)	-	2 (0.3)	> 0.05	-	-
Central venous access	1 (0.3)	1 (0.4)	-	2 (0.3)	> 0.05	-	-
Lupus anticoagulant	1 (0.3)	1 (0.4)	-	2 (0.3)	> 0.05	-	
Human Immunodeficiency Virus infection	-	1 (0.4)	1 (0.5)	2 (0.3)	-	> 0.05	-
Inherited thrombophilia	2 (0.6)	-	-	2 (0.3)	-	-	-
Factor V Leiden mutation	1 (0.3)	-	2 (1.0)	3 (0.4)	-	-	-
On bed rest or restricted mobility, including a removable leg brace for less than 72 h	1 (0.3)	-	-	1 (0.1)	-	-	-
Antiphospholipid antibodies	1 (0.3)	-	-	1 (0.1)	-	-	-
**Total Score Category**							
0 (very low risk)	-	-	-	-	-	-	-
1–2 (low risk)	28 (8.2)	11 (4.1)	3 (1.6)	42 (5.3)	0.056	0.169	**0.001**
3–4 (moderate risk)	73 (21.5)	72 (26.8)	51 (26.7)	196 (24.5)	0.128	0.988	0.217
> 5 (high risk)	239 (70.3)	186 (69.1)	137 (71.7)	562 (70.2)	0.759	0.551	0.633

p1: *P* value between pre-training and post-training period

p2: *p* value between post-training and the clinical pharmacist-intervention period

p3: *p* value between pre-training and the clinical pharmacist-intervention period

### Intervention period

During the intervention period (*n* = 191), the clinical pharmacist made a recommendation for optimal TP administration to a total of 118 patients, including 2 (66.7%) low-risk patients, 28 (55.0%) moderate-risk patients, and 88 (64.2%) high-risk patients. The acceptance rate of clinical pharmacist recommendations by surgeons was 50% in low-risk, 50% in moderate-risk, 58% in high-risk, and 56% in all patients.

### Comparison of all periods

Any TP was administered to the majority of patients at all periods (79.7%, 77.0%, and 81.2%, respectively). Any TP was administered at a rate of 65.8% in the pre-education period to patients with moderate-risk, and this rate increased to 78.4% in the clinical pharmacist-intervention period (*p* = 0.039). In high-risk patients, there was a significant increase in optimal TP rates in the clinical pharmacist-intervention period compared to the post-education period (43.5% vs. 55.5%; *p* = 0.034). When all periods were compared, the optimal TP administration rate, which was 40.6% in the pre-education period, increased to 45.4% (*p* = 0.238) in the post-education period and increased to 59.2% in the clinical pharmacist-intervention period (*p* = 0.004). On the other hand, optimal TP from the first and third periods were also compared to assess the contribution of education and clinical pharmacist interventions together, there was a significant increase in optimal TP rates for patients with high risk (36.4% vs. 55.5%, *p* = 0.001) (Table 3).

**Table 3:** Comparison of the appropriateness of the thromboprophylaxis according to the Caprini risk assessment model category

**Pre-education period (*n* = 340)**			**Post-education period (*n* = 269)**			**Intervention period (*n* = 191)**			**p1**		**p2**		**p3**	
**Category**	**Any TP, n (%)**	**Optimal TP, n (%)**	**Category**	**Any TP, n (%)**	**Optimal TP, n (%)**	**Category**	**Any TP, n (%)**	**Optimal TP, n (%)**	**Any TP**	**Optimal TP**	**Any TP**	**Optimal TP**	**Any TP**	**Optimal TP**
Low risk (*n* = 28)	13 (46.4)	13 (46.4)	Low risk (*n* = 11)	3 (27.3)	3 (27.3)	Low risk (*n* = 3)	2 (66.7)	2 (66.7)	0.471	0.471	0.505	0.505	0.600	0.600
Moderate risk (*n* = 73)	48 (65.8)	38 (52.1)	Moderate risk (*n* = 72)	44 (61.1)	38 (52.8)	Moderate risk (*n* = 51)	40 (78.4)	35 (68.6)	0.562	0.931	**0.039**	0.078	0.153	0.115
High risk (*n* = 239)	210 (87.9)	87 (36.4)	High risk (*n* = 186)	160 (86.0)	81 (43.5)	High risk (*n* = 137)	113 (82.5)	76 (55.5)	0.574	0.135	0.385	**0.034**	**0.011**	**0.001**
Total (*n* = 340)	271 (79.7)	138 (40.6)	Total (*n* = 269)	207 (77.0)	122 (45.4)	Total (*n* = 191)	155 (81.2)	113 (59.2)	0.411	0.238	0.278	**0.004**	**0.002**	**0.001**

*TP* Thromboprophylaxis

p1: *P* value between pre-training and post-training period

p2: *p* value between post-training and the clinical pharmacist-intervention period

p3: *p* value between pre-training and the clinical pharmacist-intervention period

While the rate of patients for whom the administered TP was appropriate was 46.8% in the pre-education period and 46.1% in the post-education period, this rate increased statistically significantly to 61.3% in the clinical pharmacist-intervention period compared to the post- education period (*p* = 0.001). In all classes with low, medium and high Caprini risk, the appropriateness of the selected TP method increased in the clinical pharmacist-intervention period compared to the post-education period; this increase was statistically significant in high-risk patients (44.6% and 58.4%, respectively; *p* = 0.014). While the proportion of patients for whom the selected TP method was partially appropriate was similar in the pre- and post-education periods, it decreased statistically significantly in the clinical pharmacist-intervention period compared to the post-education period (33.2%, 30% and 19.9%, respectively; *p* = 0.411, *p* = 0.014) The effect on the appropriateness of TP of patients under any TP was found to be significantly increased following education and clinical pharmacist interventions in high-risk patients (44.8% vs. 58.4%, *p* = 0.001) (Table 4).

**Table 4:** Comparison of the appropriateness of the thromboprophylaxis in patients undergoing any thromboprophylaxis

**Pre-education period (*n* = 340)**			**Post-education period (*n* = 269)**			**Intervention period (*n* = 191)**			**p1**		**p2**		**p3**	
**Category**	**Fully aTP, n (%)**	**Partially aTP, n (%)**	**Category**	**Fully aTP, n (%)**	**Partially aTP, n (%)**	**Category**	**Fully aTP, n (%)**	**Partially aTP, n (%)**	**Fully aTP**	**Partially aTP**	**Fully aTP**	**Partially aTP**	**Fully aTP**	**Partially aTP**
Low risk (*n* = 28)	13 (46.4)	-	Low risk (*n* = 11)	3 (27.3)	-	Low risk (*n* = 3)	2 (66.7)	-	0.471	-	0.505	-	0.502	-
Moderate risk (*n* = 73)	39 (53.4)	9 (12.3)	Moderate risk (*n* = 72)	38 (52.8)	6 (8.3)	Moderate risk (*n* = 51)	35 (68.6)	5 (9.8)	0.938	0.605	0.115	1.000	0.379	0.251
High risk (*n* = 239)	107 (44.8)	104 (43.5)	High risk (*n* = 186)	83 (44.6)	75 (40.3)	High risk (*n* = 137)	80 (58.4)	33 (22.1)	0.976	0.508	**0.014**	**0.002**	**0.001**	**0.001**
Total (*n* = 340)	159 (46.8)	113 (33.2)	Total (*n* = 269)	124 (46.1)	81 (30.0)	Total (*n* = 191)	117 (61.3)	38 (19.9)	0.870	0.411	**0.001**	**0.014**	**0.001**	**0.045**

*aTP* Appropriateness of thromboprophylaxis

p1: *P* value between pre-training and post-training period

p2: *p* value between post-training and the clinical pharmacist-intervention period

p3: *p* value between pre-training and the clinical pharmacist-intervention period

When optimal TP administration was evaluated according to the minor, major, and laparoscopic surgery, no significant difference was observed between the groups (*p* > 0.05). In total, it was found that the rate of optimal TP administration was lowest in patients undergoing minor surgery (23.2%), but this rate was higher in patients undergoing laparoscopic surgery (59.6%) in all periods.

According to the Caprini RAM, the rate of high-risk patients who did not receive combined TP was 30.6% in the pre-education period and 30.9% in the post-education period, and a statistically significant decrease was found as 18.3% in the clinical pharmacist-intervention period (*p* = 0.001). The rate of high-risk patients receiving mechanical prophylaxis alone, which was 27.1% in the post-education period, decreased statistically significantly to 12% in the clinical pharmacist-intervention period (*p* < 0.001). The rate of patients receiving inappropriate doses of pharmacological prophylaxis decreased significantly in the post-education period compared to the pre-education period (3.8% vs. 0.4%; *p* = 0.005). As a result, with the combined contribution of education and clinical pharmacist interventions, a significant decrease was found in the rate of high-risk patients with a single type of TP (*p* = 0.001), inappropriate duration (*p* = 0.004) and dosage of pharmacological TP (*p* = 0.018) (Table 5).

**Table 5:** Comparison of characteristics of patients not receiving optimal thromboprophylaxis

**Variables**	**Pre-education period (*n* = 340)**	**Post-education period (*n* = 269)**	**Intervention period (*n* = 191)**	**Total (*n* = 800)**	**p1**	**p2**	**p3**
High-risk patients with a single type of TP	104 (30.6)	83 (30.9)	35 (18.3)	222 (27.8)	0.628	**0.001**	**0.001**
*Only mechanical TP*	95 (27.9)	73 (27.1)	23 (12.0)	191 (23.9)	0.327	** < 0.001**	** < 0.001**
*Only pharmacological TP*	9 (2.6)	10 (3.7)	12 (6.2)	31 (3.9)	0.819	0.886	**0.009**
Not receiving any TP	69 (20.3)	62 (23.0)	36 (18.8)	167 (20.9)	0.259	0.205	0.688
Inappropriate duration of pharmacological TP	13 (3.8)	3 (1.1)	1 (0.5)	15 (1.9)	0.064	0.761	**0.004**
Moderate risk patients who receive combined TP	9 (2.6)	6 (2.2)	5 (2.6)	20 (2.5)	0.605	0.622	0.889
Inappropriate dosage of pharmacological TP	13 (3.8)	1 (0.4)	3 (1.5)	17 (2.1)	**0.005**	0.356	**0.018**
Total	202 (59.4)	147 (54.6)	78 (40.8)	427 (53.4)	0.238	**0.004**	**0.002**

*TP* Thromboprophylaxis

p1: *P* value between pre-training and post-training period

p2: *p* value between post-training and the clinical pharmacist-intervention period

p3: *p* value between pre-training and the clinical pharmacist-intervention period

A total of 124 patients undergoing abdominal-pelvic cancer surgery were identified as high-risk, of whom 81.5% administered optimal TP and 29% administered prolonged (post-discharge) TP lasting more than 28 days.

### Post-operative VTE & major bleeding

During the 30-day follow-up period, VTE was observed in 4 (1.2%) patients in the pre-education and 1 (0.4%) patient in the post-education period. No VTE was observed in any patient in the clinical pharmacist-intervention period. In the pre-education period, it was determined that the risk of VTE was high in 3 patients (inferior vena cava thrombosis, DVT, and PTE), moderate in 1 patient (portal vein thrombosis) and optimal TP was administered to all of these patients. Major post-operative bleeding was observed in 6 (1.8%) patients in the pre-education period, 5 (1.9%) patients in the post-education period, and 2 (1.0%) patients in the clinical pharmacist-intervention period. It was determined that pharmacological prophylaxis was not administered in 1 of 13 patients with major bleeding and this patient was in the post-education period. In 1 of the 2 patients with major bleeding in the clinical pharmacist-intervention period, it was observed that the clinical pharmacist's recommendation was not implemented and high-dose pharmacological prophylaxis treatment was continued.

## Discussion

In this study, VTE risk and prophylaxis in hospitalized and operated patients in a tertiary referral hospital were evaluated in pre-education, post-education, and clinical pharmacist-intervention periods. Although there are studies in the literature [8–10] evaluating the contribution of education or clinical pharmacist’s intervention for TP administration, this is the first study evaluating the contribution of clinical pharmacist in addition to the improvement after education using Caprini RAM.

According to the DissolVE-2 study in which TP was evaluated, the most common statements of Caprini RAM were major surgery (52.6%), 41–60 years of age (45.4%), cancer (27.5%), laparoscopic surgery (23.7%), minor surgery (21.5%), and BMI > 25 kg/m^2^ (19.6%) [11]. In our study, the rates of 41–60 years of age (45.6%) and laparoscopic surgery (24.1%) were similar. However, the rates of major surgery (64.0%), BMI > 25 kg/m^2^ (67.1%) and cancer (43.4%) were higher. It is estimated that due to the COVID-19 pandemic, minor surgeries were postponed and complicated surgeries were performed at a relatively higher rate as our hospital is one of the largest tertiary referral hospitals.

In our study, the higher rate of patients with high-risk of VTE (70.2%) compared to the literature [11–13] may be explained by the fact that Caprini RAM 2013, which includes additional risk factors, was used to evaluate the risk of VTE. Caprini RAM 2013 was preferred to be used because current literature indicate that Caprini RAM 2013 version is more accurate and effective method for VTE risk assessment compared with Caprini RAM 2009 [14]. Also, the rate of patients undergoing any TP in all periods was found to be higher (77.0 to 81.2%) compared to the current literature [2, 15–17]. This difference may be due to the fact that the other studies were retrospective and conducted with different ethnic groups. In addition, the high rate of complicated cases being operated may have changed the TP attitudes of general surgeons and increased the rate of any TP administration. In line with these findings, it was concluded that the TP awareness of general surgeons in our hospital was high.

The rate of moderate-risk patients who administered any TP increased to 78.4% in the clinical pharmacist-intervention period, resulting in a significant improvement compared to the education-based literature [12]. There is no study examining the rate of any TP administration and the effect of clinical pharmacist in operated patients with moderate-risk. In our study, it was shown that although surgeons have a high awareness of any TP administration in high-risk patients, it may be overlooked in moderate-risk patients, and therefore, with the involvement of the clinical pharmacist in follow-up, surgeons' TP administration in moderate-risk patients may improve.

Studies have shown that the rate of patients who did not receive any TP in surgical patients was 28.1–77.5% [16–19]. In a study which compliance with TP guidelines of multifaceted interventions including reminder was evaluated, it was found that the rate of patients with VTE risk for whom prophylaxis was neglected was 45% before the intervention and it was decreased to 13.3% after the intervention (*p* < 0.001) [20]. Although not statistically significant, the rate of patients who were not given any TP was decreased with the clinical pharmacist intervention compared to the post-education period. These results support the importance of the clinical pharmacist involvement in the implementation and quality of TP in surgical wards.

According to the 9^th^ ACCP guideline, we found that inappropriate dosage of pharmacological prophylaxis (2.1%) decreased significantly with education (*p* = 0.005). In two studies, in which compliance with the 6^th^ and 7^th^ ACCP guidelines between 2008 and 2009 was examined in TP administration, the rate of patients receiving inappropriate doses of pharmacological prophylaxis was 9.3% and 10.9%, respectively [17, 19]. The lower rate of dose inappropriateness in our study compared to the literature may be due to increased awareness of VTE guidelines, knowledge, and experience of surgeons.

Although many guidelines recommend prolonged TP in high-risk patients undergoing abdominal pelvic cancer surgery, in our study, the rate of these patients who received the ACCP-recommended TP was found to be low (29%).

The use of intermittent pneumatic compression (IPC) devices for VTE prophylaxis is appropriate when used according to the latest clinical recommendations [21]. In our study, we observed that IPC devices, which are recommended by guidelines as first-line mechanical prophylaxis, were largely underutilized in patients. This may be due to various reasons such as insufficient awareness of its role in TP, insufficient number of devices, hospital management problems and errors of omission. In this context, it is considered important to develop strategies to increase the use of IPC devices.

The important limitations of our study are that the education was not repetitive, the risk assessment was performed only in the pre-operative period, and the inadequacy of the IPC devices.

## Conclusion

In our study in which VTE risk and prophylaxis were evaluated, inappropriateness in optimal TP administration were pointed out. In operated patients, it is important to consider the Caprini RAM and guidelines in the process of assessing the risk of VTE and making the optimal TP decision. In our study, it was demonstrated that interventions and education resulted in an improvement in optimal TP. The results of our study need to be supported by larger prospective randomized controlled trials with the implementation of clinical pharmacy services.

## Data Availability

The data presented in this study are available on request from the corresponding author. The data are not publicly available due to restrictions privacy and ethical.
